# Predicting time across age: comparing performance and neural dynamics of younger and older adults in a temporal prediction task

**DOI:** 10.3389/fnagi.2026.1790156

**Published:** 2026-05-20

**Authors:** Marleen J. Schoenfeld, Rebecca Burke, Till R. Schneider, Andreas K. Engel

**Affiliations:** 1 Department of Neurophysiology and Pathophysiology, University Medical Center Hamburg-Eppendorf, Hamburg, Germany; 2 Hamburg Center of Neural and Cognitive Systems, University Medical Center Hamburg-Eppendorf, Hamburg, Germany

**Keywords:** ageing, beta activity, delta inter-trial phase consistency, electroencephalography, temporal prediction

## Abstract

Temporal prediction is the ability to anticipate the likely time of occurrence of events and is important for adaptive behaviour in our everyday lives. Studies indicated that predictive aspects of environmental stimuli can be leveraged to reduce reaction times and enhance stimulus processing. Particularly, a recent study showed that this optimized behaviour is associated with a phase adjustment of ongoing neural oscillations aligning with the expected onset of upcoming stimuli. In ageing, there is some evidence that temporal predictions might be altered, however findings are inconsistent. Thus, we aimed to explore whether and how anticipatory oscillatory markers of temporal prediction change with age. To this end, 20 right-handed younger adults (14 females, age = 25.4 ± 4.13 years) and 20 right-handed older adults (13 females, age = 60.35 ± 3.92 years) performed a temporal prediction task while 64-channel EEG was recorded. In the task, a visual stimulus moved across the screen toward an occluder, disappeared and reappeared after a variable time delay. Participants judged whether the visual stimulus reappeared “too early” or “too late.” Results indicated comparable temporal prediction performance between younger and older adults. We observed a stronger task-related decrease of beta activity in older compared to younger adults. Further, we observed phase adjustments of ongoing delta activity aligning with the disappearance of moving stimuli which was stronger in older compared to younger adults. Together, these observations provide several new insights into the neural dynamics supporting temporal prediction and suggest that neural dynamics associated with temporal prediction appear sensitive to ageing.

## 1 Introduction

Temporal prediction is the ability to anticipate when events are likely to occur (Coull and Nobre, 1998) and is crucial for adaptive behaviour in everyday life, as it enables optimized behaviour such as faster reaction times (Lakatos et al., 2008; Rohenkohl and Nobre, 2011) and enhanced stimulus processing (Wilsch et al., 2015).

Neural oscillations, i.e., frequency- and phase-specific fluctuations of neuronal activity, are thought to enable flexible communication and information processing between neuronal populations (Engel et al., 2001; Senkowski and Engel, 2024). In temporal prediction, this is typically associated with activity in the alpha (8–14 Hz) and beta (13–30 Hz) frequency band (van Ede et al., 2011) as well as phase alignment in the delta frequency band (Arnal et al., 2015; Schroeder and Lakatos, 2009). Specifically, the attenuation of alpha activity is associated with the onset (Rohenkohl and Nobre, 2011) and location (Thut et al., 2006) of anticipated visual stimuli. A decrease of alpha activity is interpreted to reflect enhanced cortical excitability (Sauseng et al., 2005), while increases of alpha activity are potentially reflecting an active inhibitory process (Jensen and Mazaheri, 2010). Thus, alpha activity is thought to indicate when in time and how much attention is deployed (Herrmann et al., 2023). A decrease in beta activity is associated with the onset of a predicted stimulus (but also see Gulberti et al., 2015) and linked to optimized response behaviour (Arnal et al., 2015; Daume et al., 2021). Further, the decrease of beta activity has been reported to indicate the involvement of sensorimotor areas (Morillon and Baillet, 2017) and task-relevant sensory regions (Daume et al., 2021; van Ede et al., 2011). Thus, beta activity is interpreted to signal preparatory processes in the sensory system that expects the upcoming event (Engel and Fries, 2010). Next to the activity in the alpha and beta band, neural activity in the delta (1–4 Hz) frequency band is reported to entrain, i.e., temporally align in phase, to expected stimuli and thereby enable temporal prediction (Arnal et al., 2015; Schroeder and Lakatos, 2009). Daume et al. (2021) reported a phase adjustment of ongoing neural oscillations in the delta frequency band aligning with the expected onset of upcoming stimuli in a non-rhythmic temporal prediction task. Supporting this finding, the application of delta transcranial alternating current stimulation (tACS) led to a phase-dependent modulation of performance in a similar task, demonstrating that entrained oscillatory dynamics can influence performance phase-specifically (Burke et al., 2025). Thus, neural oscillations seem to play a key role in determining whether an event occurs at the expected time, most likely due to power modulations in higher frequency bands and a phase adjustment of ongoing neural oscillations in lower frequency bands.

In ageing, behavioural and neurophysiological functions underlying temporal predictions might be altered; however, findings are inconsistent (see review Zanto and Gazzaley, 2014). Typically, ageing is associated with a decline in processing speed (Salthouse, 1996), executive control and other cognitive abilities (Lindenberger, 2014; Salthouse, 2010, 2011). Several studies reported reduced timing abilities in older adults (Mioni et al., 2020; Zanto et al., 2011). In contrast, other studies indicated that allocating attention to moments in time is preserved in older adults (Chauvin et al., 2016; Shalev et al., 2024). Heideman et al. (2018) reported that the ability to allocate attention was associated with stronger anticipatory modulation of alpha and beta activity in older compared to younger adults. Thus, it remains inconclusive whether and how ageing alters temporal prediction and its underlying neural dynamics.

In this study, we aimed to investigate whether anticipatory oscillatory markers of temporal prediction vary with age using an established temporal prediction task (Burke et al., 2025, 2026; Daume et al., 2021), while recording electroencephalography (EEG) in younger and older adults. We hypothesized that when comparing the two age groups, younger adults show better behavioural performance, i.e., more accurate temporal prediction, than older adults. Based on Daume et al. (2021), we hypothesized to observe power modulations in the beta frequency band while predicting the likely occurrence of stimuli and that these modulations differ between groups. Further, we hypothesized to observe phase consistency in the delta frequency band following stimulus offset, and that phase consistency differs between groups. In addition, we expected the neural data to correlate with temporal prediction performance such that more accurate temporal prediction is associated with stronger power modulations in the beta frequency band or phase consistency in the delta frequency band.

## 2 Materials and methods

### 2.1 Participants

In this study, 20 right-handed healthy younger adults (14 females, age = 25.4 ± 4.13 years, range 20–35 years, education = 16.15 ± 2.18 years) and 20 right-handed healthy older adults (13 females, age = 60.35 ± 3.92 years, age range 56–69 years, education = 12.75 ± 2.95 years) gave their written informed consent to take part in the study, which was conducted in accordance with local ethics committee approval (Ethik-Kommission der Ärztekammer Hamburg, PV7102), and the Declaration of Helsinki. The number of participants was based on experience and previous studies using this task (Burke et al., 2025; Daume et al., 2021). All participants were monetarily compensated for taking part in the study. All had normal or corrected-to-normal vision and reported no history of neurological or psychiatric disorders. Handedness was assessed with the short version of the Edinburgh Handedness Inventory (Oldfield, 1971). While the present manuscript focuses on age-related differences between younger and older adults, data for older adults are also included in a separate publication comparing older adults with Parkinson’s disease patients (Burke et al., 2026).

### 2.2 Experimental design

**Figure 1:**
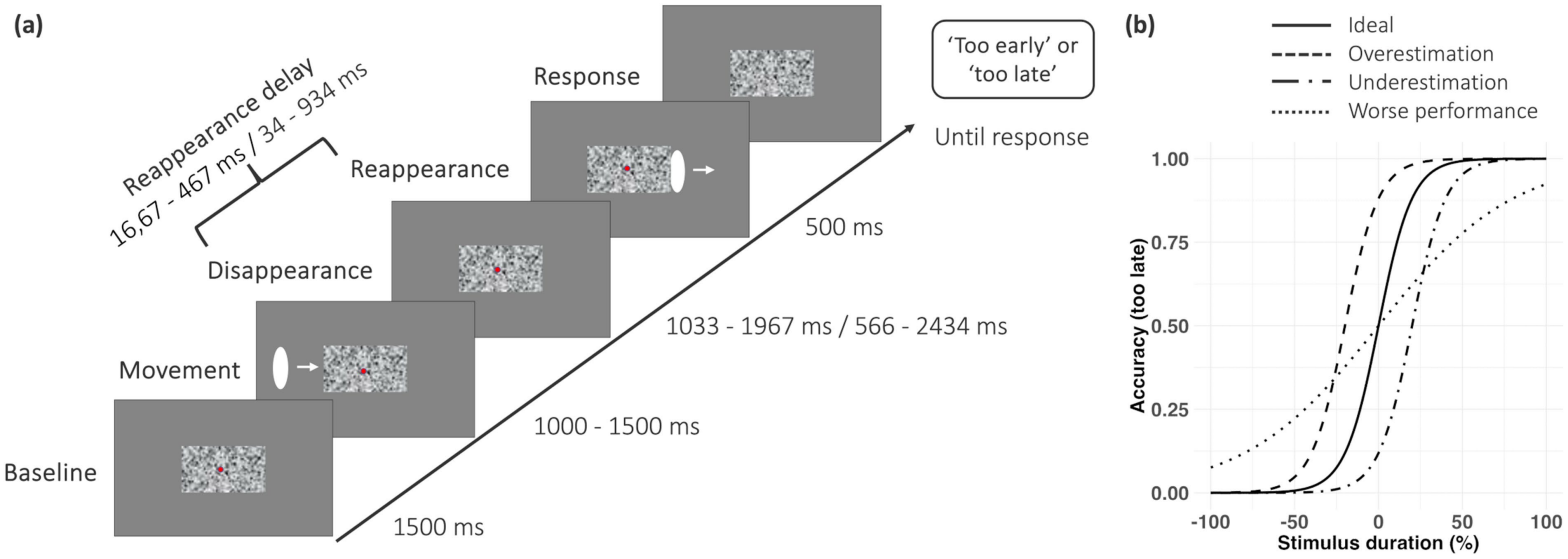
Experimental design and trial structure. **(a)** Each trial started with a baseline. After baseline, a visual stimulus (white ellipse) appeared on the left side of the screen, moved toward the centre of the screen and disappeared behind an occluder (grey rectangle). After the reappearance delay, the visual stimulus reappeared, and participants were asked to judge whether the stimulus reappeared “too early” or “too late” in relation to its prior movement. The reappearance delay differed between conditions (original condition: 17–467 ms in steps of 50 ms; simplified condition: 34–934 ms in steps of 100 ms). Each participant performed 480 trials in each condition. **(b)** Example of behavioural performance patterns in the task. Performance on the temporal prediction task is often visualised using a psychometric curve, which plots the proportion of “too late” responses against stimulus duration. An ideal curve (solid) depicts performance of a subject who always classified early reappearance times as “too early” and late reappearance times as “too late,” with equal likelihood at 0 ms. A leftward shift (dashed) indicates overestimation, while a rightward shift (dotted-dashed) suggests underestimation of the time delay. A flatter curve (dotted) reflects poorer temporal prediction performance, with “too early” responses to late reappearance times and vice versa.

The study was conducted in two sessions on separate recording days with a maximum of 1 week apart. In the beginning of each session, participants performed neuropsychological tests, specifically the Trail Making Test A and B (Reitan, 1956) and digital version of Berg’s Card Sorting Test (Grant and Berg, 1948) to determine whether they were cognitively fit to take part in the study (see Table 1). Participants who did not pass either of the tests were excluded from the study. Next, the temporal prediction task was performed; for details of the task see Daume et al. (2021). Participants were positioned in a low-light environment inside an electrically insulated, soundproof chamber designed to minimize external interference. Each trial started with a baseline period of 1,500 ms. Following baseline, a visual stimulus (white ellipse) moved across the screen, disappeared behind an occluder (white noise rectangle) for a varying period of time, i.e., reappearance delay, and reappeared (Figure 1a). The visual stimulus moved from the left side to the right side of the screen with constant velocity and its movement onset was jittered across trials (1,000, 1,100, 1,200, 1,300, 1,400 or 1,500 ms) relative to the objectively correct 1,500 ms interval. During disappearance, participants predicted the time of when the visual stimulus would reappear again, thus, this was the specific time of interest in the experiment. When the visual stimulus reappeared for 500 ms, participants had to judge whether it appeared “too early” or “too late” based on the speed of the stimulus before disappearance. Participants indicated their response by pressing a corresponding button on a button box with their right index finger. Which button corresponded to which response (i.e., response mapping) was counterbalanced across participants.

**Table 1:** Results of the neuropsychological tests for younger and older adults.

Parameter	Younger adults	Older adults	Paired-samples *t*-tests *p* value
Trial making Test A			
Time (s)	23.49 ± 6.57	33.53 ± 11.4	**0.005**
Percentile	46.47 ± 23.96	55 ± 24.77	0.679
Trial making Test B			
Time (s)	66.97 ± 26.15	83.51 ± 24.1	0.06
Percentile	60 ± 23.09	45.88 ± 26.71	0.42
Berg’s card sorting test			
Total errors	17.69 ± 4.62	24.79 ± 10.23	**0.016**
Percentile	52.3 ± 19.63	56 ± 29.15	0.688
Perseverative errors	10. 46 ± 2.44	15.61 ± 5.83	**0.003**
Percentile	40.25 ± 20.87	46.8 ± 23.28	0.435

Data are given as mean ± SD. Bold values mean the *p*-values are significant.

The task was performed with two conditions, an original condition (Daume et al., 2021) and a simplified condition. In the simplified condition, the task structure was identical to the standard condition. However, the range of reappearance delays was expanded, resulting in reduced temporal uncertainty and a subjectively less complex task experience. To simplify judgements, the reappearance delays were increased from 17–467 ms in the original condition (randomly varied from ±17 to ±467 ms, in steps of 50 ms) to 34–934 ms in the simplified condition (randomly varied from ±34 to ±934 ms, in steps of 100 ms) relative to the objectively correct 1,500 ms interval. Thus, the stimulus was covered by the occluder for 1,033–1,967 ms in the original condition and 566–2,434 ms in the simplified condition and was reappearing at 20 different time points in each condition.

In each session, 8 blocks, i.e., four blocks per condition, were completed and each block contained 60 trials. This resulted in a number of 480 trials in each session and condition, and a total of 960 trials across sessions. The order of conditions was pseudorandomized across participants and sessions, i.e., in each session 4 blocks of each condition were presented. At the end of each block, participants were informed about their performance by presenting their overall accuracy as a percentage. The start of each block was self-paced; thus, participants could decide on their own when to start the next block. In the beginning of each session, the task was practiced for an additional 30 trials for familiarisation. Depending on the response times of participants, the task was completed in 50–60 min. Throughout the task, participants were instructed to fixate a red dot in the centre of the occluder. Once participants gave their response, the fixation dot turned dark grey for 100 ms indicating participants’ response was registered. Fixation was trained during familiarisation trials and visually monitored by the experimenters as eye movements are visible in the EEG recording. We specifically did not use eye-tracking as the study by Daume et al. (2021) reported that the results in the neural data were not explained by horizontal eye movements during temporal predictions.

The task was implemented using MATLAB R2016b version 9.1.0.441655 (The MathWorks Inc., Natick, MA, United States; RRID: SCR_001622) and Psychtoolbox version 3.0.13 (Brainard, 1997) (RRID:SCR_002881). Visual stimuli were presented with a refresh rate of 120 Hz and 1,920 × 1,080 pixels positioned 50 cm in front of the participants. Participants wore earplugs at all times to avoid distractions.

### 2.3 Behavioural data analysis

**Figure 2:**
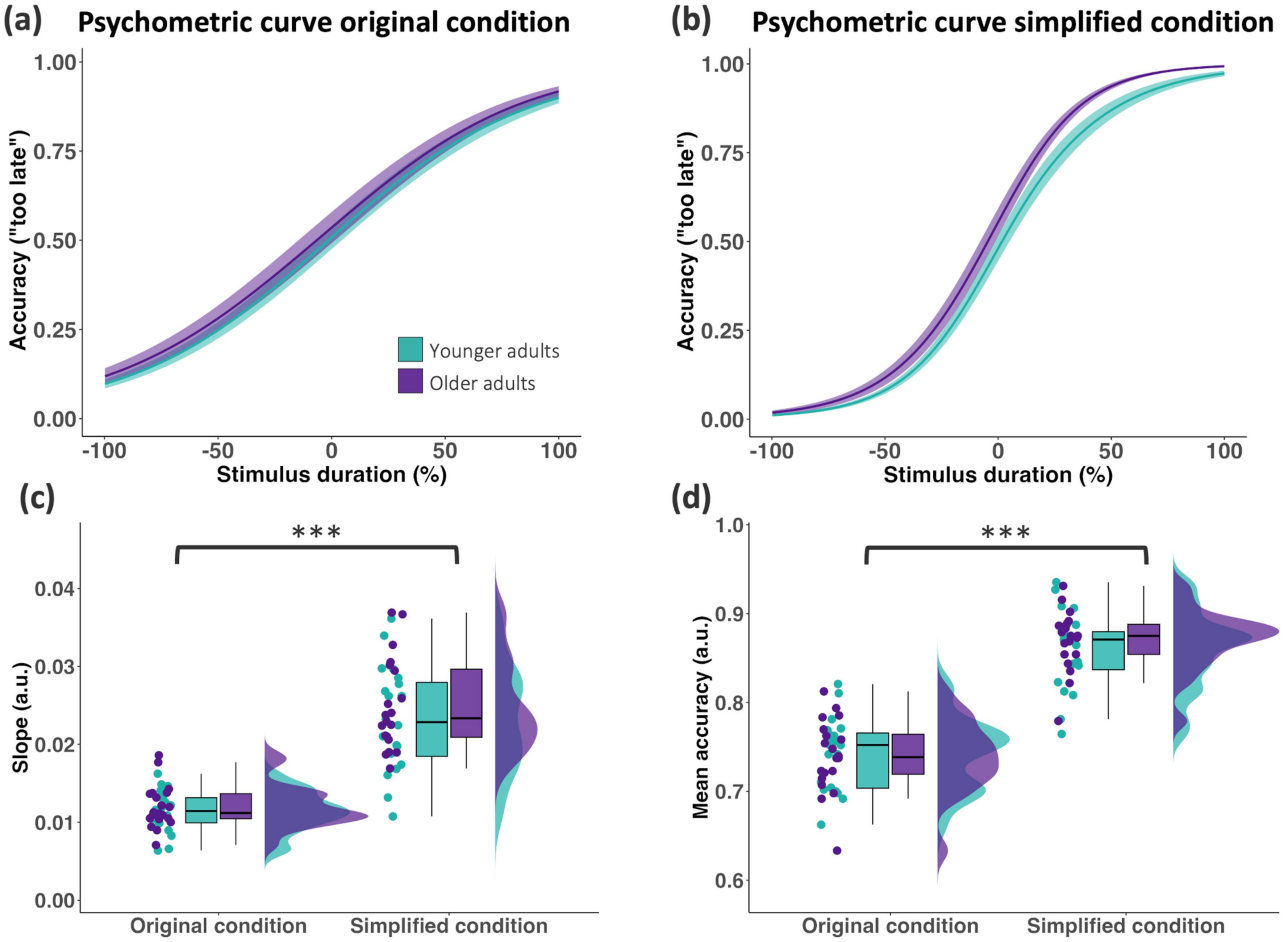
Comparable performance in the temporal prediction task for younger (green) and older (purple) adults (each group *N* = 20). Performance for “too late” responses were used to determine the psychometric curve for **(a)** the original condition and for **(b)** the simplified condition of the temporal prediction task. **(c)** Slope of the psychometric curve was averaged across original (left) and simplified (right) condition in each group. Data are shown on single subject level, boxplot, and distribution (each *N* = 20). **(d)** Mean accuracy was averaged across original (left) and simplified (right) condition in each group. Data are shown on single subject level, as boxplots, and as distributions (*N* = 20). ***p < 0.001.

To determine participants’ temporal prediction performance in the task, we fitted a psychometric curve for each condition and group using binomial logistic regression (Figure 1b). For each timing difference, i.e., delay in the reappearance of the visual stimulus relative to its expected time, the proportion of “too late” answers was computed for each participant (Figures 2a,b). The fit of the psychometric function was calculated using the *glmfit.m* and *glmval.m* functions provided in MATLAB. The fitted timing difference value at 50% proportion “too late” answers was defined as the subjective “right on time” for each participant, as this point reflects the timing difference at which participants are equally likely to judge the stimulus as “too early” or “too late.” This measure was used to test for a potential bias toward “too early” or “too late” responses. Slope, i.e., steepness of the psychometric function, was computed as the reciprocal of the difference between fitted timing difference values at 75 and 25% proportion “too late” answers. Mean accuracy was computed as the average percentage of correct responses, i.e., “too late” response in a trial in which the visual stimulus reappeared too late. Response times were measured from the time point of stimulus reappearance until the participant’s key press. Behavioural data were visualised using R (version 4.3.1; RRID: SCR_001905) (R Core Team, 2023) and RStudio (Version 2023.06.1; RStudio Inc., Boston, USA; RRID: SCR_000432).

### 2.4 Statistical analysis

To investigate differences between younger and older adults, the slope, mean accuracy and response times were averaged across participants for each condition and group separately. Normality was assessed using Shapiro–Wilk tests. For repeated-measures analyses, sphericity was assessed using Mauchly’s test. Single subject data was screened for outliers, and no extreme outliers requiring exclusion were identified. Data were analysed using paired samples *t*-tests with Bonferroni correction for multiple comparisons, and repeated measures analysis of variance (ANOVA). Significant interactions were followed up using paired samples *t*-tests with Bonferroni correction for multiple comparisons. Correlations were assessed using Pearson’s correlation. All reported *p*-values for *t*-tests were two-tailed and the significance level was set at *p* < 0.05. Statistical analyses were performed using the opensource software JASP (version 0.18.3; RRID: SCR_015823) (JASP Team, 2024). Please see the section below for the statistical analysis of the EEG data.

### 2.5 EEG data analysis

#### 2.5.1 Data acquisition and pre-processing

While the temporal prediction task was performed, EEG was continuously recorded at a sampling rate of 1,000 Hz using a 64-channel gel-based active electrode system with actiCAP slim electrodes and a BrainAmp amplifier (Brain Products GmbH, Gilching, Germany) in a sound attenuated and electrically shielded room. All sites were referenced to FCz and grounded to Fpz and positioned according to the international 10–10 system.

For data analysis, we followed a similar protocol as Daume et al. (2021). EEG Data were analysed using MATLAB R2020a version 9.8.0.1323502 (RRID: SCR_001622) with FieldTrip version 20210928 (Oostenveld et al., 2011) (RRID:SCR_004849), and the MATLAB-based toolbox “MEG and EEG Toolbox of Hamburg” (METH toolbox, Guido Nolte, version April 15, 2019; RRID: SCR_016104). EEG data was first cut into epochs to only contain time on task and consequently, each trial was cut 1,250 ms earlier to stimulus movement onset and 1,250 ms after offset of the reappearing stimulus. Trials containing strong muscle artefacts or jumps were detected by semi-automatic procedures implemented in FieldTrip and data above a threshold (*z*-value of 20) were excluded from analysis. Slow drifts, high frequency noise and line noise were removed by applying a bandpass filter at 1–100 Hz, and a band-stop filter (49–51 Hz). Subsequently, data were down-sampled to 500 Hz to reduce computational demands. Further artefact removal was performed with Independent Component Analysis (ICA). Independent components that represented stereotypical artefacts such as eye blinks, eye movements, and electrical heartbeat activity were manually identified and removed by back-projecting all but these components. On average, 4.7 ± 0.9 components of a total of 64 components were rejected in each participant and each session. Thus, 459.4 ± 17.19 trials of the total of 480 trials remained from pre-processing on average across sessions and participants.

#### 2.5.2 Time-frequency analysis

For all analyses of the EEG data, we only considered correct trials. This decision was based on two considerations. First, our behavioural data showed that participants’ subjective judgements of a stimulus reappearing “right on time” were not systematically biased toward either “too early” or “too late” responses. This suggests that objectively correct trials were also generally perceived as subjectively correct (see behavioural data analysis). Second, similar findings were reported by Daume et al. (2021), who observed comparable results for both subjectively and objectively correct trials (see their paper for full description). For spectral analysis, we decomposed the EEG recordings into time-frequency representations by convolving the data with complex Morlet’s wavelets (Cohen, 2014). The recording of each trial and channel was convolved with 30 complex wavelets, logarithmically spaced between 1 and 60 Hz. With increasing frequency, the number of cycles for each wavelet logarithmically increased from 2 to 10 cycles. Due to variations in timing differences between the movement onset, disappearance behind the occluder and reappearance of the stimulus across trials, averaging would heavily smear the power estimates of events within each trial. Therefore, spectral power estimates were computed for four distinct, partially overlapping time windows aligned to the respective events (see Figure 1a): a “baseline” window (−550 to −50 ms before movement onset); a “movement” window (−50 to 950 ms relative to movement onset); a “disappearance” window (−350 to 950 ms relative to stimulus disappearance); and a “reappearance” window (−350 to 450 ms relative to stimulus reappearance). To maintain consistent time-frequency representations, trials were segmented after wavelet convolution. Spectral power estimates were averaged across all trials within each window, 100 ms time bins (centred on each full deci-second) and normalised using the pre-stimulus baseline window (−500 to −200 ms before movement onset, normalised as percentage change). To assess data quality of the spectral power modulations associated with trial events, power estimates were averaged across channels to obtain a grand average for each group (separate grand average for each condition). Statistical analysis was conducted by comparing each time-frequency pair of the movement, disappearance and reappearance windows against the pre-stimulus baseline using paired-sample *t*-tests. To account for multiple comparisons, cluster-based permutation statistics were applied (cluster-*α* = 0.05, 1,000 randomisations), as implemented in FieldTrip (Maris and Oostenveld, 2007). Subsequent analysis was focused on the disappearance window for two reasons. First, during the disappearance window participants were asked to predict the occurrence of the visual stimulus and was therefore our primary time of interest. Second, the movement, as well as the reappearance of the visual stimulus were jittered with respect to the disappearance of the stimulus. This strongly reduced their effect on power estimates computed during the disappearance window and therefore impacted interpretability. Given our primary interest in age-related differences during temporal prediction, we focused our analyses on spectral power estimates in the disappearance window within those frequency bands that exhibited significant deviations from baseline in the grand average comparison. These estimates were then compared between younger and older adults to assess age-related differences. As the disappearance window showed significant power differences between groups exclusively in the beta band (13–30 Hz), we conducted subsequent analyses within this frequency range. Statistical tests were performed at each time point within the disappearance window across all channels, comparing groups for each condition separately, using cluster-based permutation statistics. For source-level analysis, we employed the METH toolbox that implements a single-shell volume conductor model (Nolte, 2003) with 5,003 grid points aligned to the MNI152 template brain. Cross-spectral density (CSD) matrices were computed from wavelet-transformed data in 100 ms steps within the predefined time windows and used to compute common adaptive linear spatial filters (DICS beamformer; Gross et al., 2001). Source power was normalised to the pre-stimulus baseline. Age-related differences between groups in beta power during the disappearance window were identified using cluster-based permutation statistics (separately for each condition). Differences across conditions in beta power during the baseline and disappearance window were also assessed using cluster-based permutation statistics (separately for each group). To examine neural-behavioural associations, we computed voxel-wise Pearson correlations between beta power and individual psychometric function slope, averaging power estimates from −0.3 to 0.7 s in the beta range and correcting for multiple comparisons through cluster-based permutation statistics. A similar correlation using mean accuracy instead of the slope of the psychometric function, as well as a pooled correlation across all groups and conditions (*N* = 80), was performed.

#### 2.5.3 Inter-trial phase consistency

To quantify phase alignments, we measured inter-trial phase consistency (ITPC), using the complex time-frequency representations obtained from the wavelet convolution, as described above. Specifically, the phase of the complex data was extracted for each time point and trial (with function *angle.m* in MATLAB). ITPC was then computed across all correct trials within each of the four time-windows in all frequencies (for formula and full description, see Cohen, 2014). ITPC can vary between 0 and 1; 0 means no phase consistency across trials and 1 means all trials show the same phase. Once computed, ITPC estimates were averaged across trials and averaged in 100 ms bins across the four time windows (see above). Similar to the spectral power analysis above, we first obtained a general overview of ITPC estimates associated with the different trial events. Statistical analysis was conducted by comparing ITPC estimates of the movement, disappearance and reappearance windows against the pre-stimulus baseline using paired-sample *t*-tests accounting for multiple comparisons with cluster-based permutation. As described above, subsequent analyses focused on the disappearance window, because this was our primary time of interest and because jittered movement and reappearance of the visual stimulus relative to disappearance smears effects in the ITPC estimates, thereby limiting the interpretability of the results. Given our primary interest in age-related differences in phase consistency in the delta frequency band during temporal prediction (based on findings by Daume et al., 2021), subsequent analyses focused on delta (1–4 Hz) ITPC during the disappearance window. We conducted cluster-based permutation statistics for delta ITPC to identify clustering channels and timepoints that showed statistically significant differences between the groups. For source-level analysis, we computed ITPC using the same steps as described for source-level spectral power. To assess age-related group differences in delta ITPC during the disappearance window, cluster-based permutation statistics were employed (separately for each condition). To examine differences across conditions in delta ITPC during the disappearance window, cluster-based permutation statistics were used (separately for each group). To examine whether the behavioural and neural data correlated, we conducted Pearson correlations with delta ITPC and individual slopes of the psychometric function. For this analysis, we averaged delta ITPC estimates of significant voxels from −0.3 to 0.6 s and accounted for multiple comparisons using cluster-based permutation statistics. We performed the same correlation with mean accuracy instead of slope. To increase statistical power, we also conducted a pooled correlation using data from all groups and conditions (*N* = 80).

## 3 Results

### 3.1 Comparable temporal prediction performance in younger and older adults

To compare the temporal prediction performance in the task across the two age groups, a 2 × 2 ANOVA was conducted with *group* (younger, older adults) as a between-subject factor, *condition* (original, simplified) as a within-subject factor, and either the slope of the psychometric curve or mean accuracy as the dependent variable. For the dependent variable slope, we found a significant main effect of *condition* (*F*_1,19_ = 245.224, *p* < 0.001, *η*^2^*_p_* = 0.928; Figure 2c), indicating that task simplification influenced participants’ response patterns. Likewise, for the dependent variable of mean accuracy, we found a significant main effect of *condition* (*F*_1,19_ = 1321.021, *p* < 0.001, *η*^2^*_p_* = 0.986; Figure 2d), demonstrating enhanced accuracy in the simplified condition. Notably, no interactions or significant effects for *group* were found (all *p* > 0.05), suggesting that while the simplified condition resulted in a steeper slope and higher mean accuracy, performance remained comparable between younger and older adults. This implies that while task difficulty influenced temporal prediction sensitivity, aging did not significantly alter temporal prediction performance.

To test whether participants exhibited a significant bias toward responding “too early” or “too late” in the temporal prediction task, we performed 4 one-sample *t*-tests against zero, using each participant’s “right on time” estimate as the dependent variable. Across participants, there was no significant bias in either younger adults (original condition: *t*(19) = −0.038, *p* = 0.970, Cohen’s *d* = −0.008; simplified condition: *t*(19) = 1.301, *p* = 0.209, Cohen’s *d* = 0.291) or older adults (original condition: *t*(19) = −1.01, *p* = 0.325, Cohen’s *d* = −0.226; simplified condition: *t*(19) = −1.793, *p* = 0.089, Cohen’s *d* = −0.401). In addition, we tested whether response bias differed between age groups by computing a 2 × 2 ANOVA with a between-subject factor *group* (younger, older adults) and a within-subject factor *condition* (original, simplified) for the dependent variable of “right on time.” Results showed no significant difference across groups or conditions (all *p*’s > 0.05, Supplementary Figure 1). Overall, these findings indicate that participants did not systematically favour either a “too early” or “too late” response, suggesting an absence of response bias in temporal prediction across age groups.

### 3.2 Response times were slower in older compared to younger adults

To investigate whether response times varied across age groups, we performed a 2 × 2 ANOVA with the dependent variable ‘response time’ and between-subject factor *group* (younger, older adults) and within-subject factor *condition* (original, simplified). The analysis revealed a significant main effect of *group* (*F*_1,19_ = 7.058, *p* = 0.016, *η*^2^*_p_* = 0.271), indicating that older adults responded slower than younger adults. Additionally, a significant main effect of *condition* (*F*_1,19_ = 24.288, *p* < 0.001, *η*^2^*_p_* = 0.561) suggested that response times were faster in the simplified condition compared to the original condition. However, the interaction between *group* and *condition* was not significant (*p* > 0.05, see Supplementary Figure 2), indicating that both groups benefited similarly from the simplified condition. These findings highlight age-related slowing in response execution while also demonstrating that task simplification improved response efficiency across groups.

### 3.3 Stronger decrease in beta activity in older compared to younger adults in original condition

**Figure 3:**
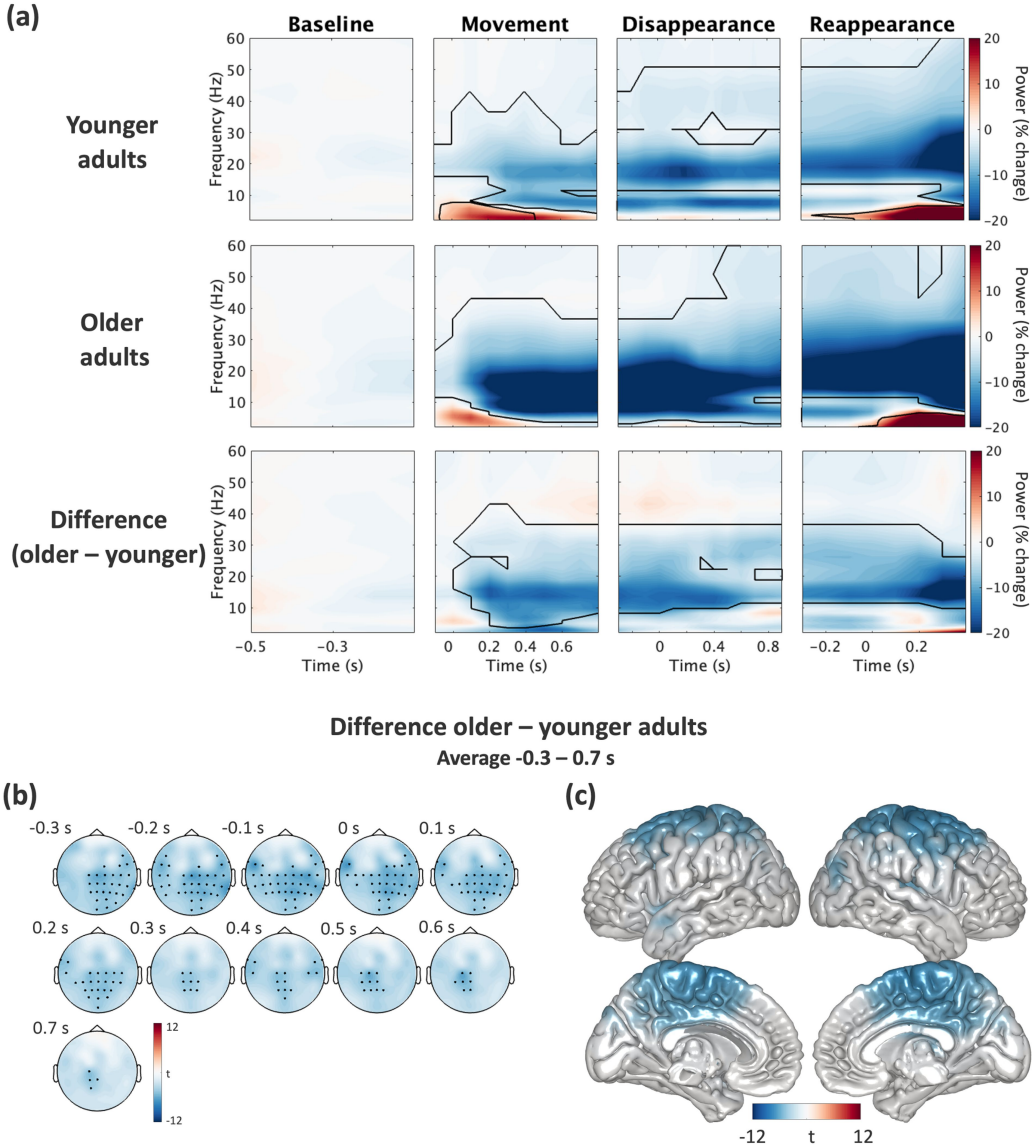
Grand-average power modulation in the original condition of the temporal prediction task. **(a)** Spectral power dynamics in the original condition are shown separately for younger adults, older adults, and the difference between both groups (older-minus-younger adults) during the baseline, movement, disappearance, and reappearance window of the temporal prediction task. For each window, epochs were aligned to the respective events (time 0 s). Spectral power was baseline-corrected within each group. Cluster-based permutation statistics revealed significant power modulations as compared to baseline (highlighted by continuous lines) and significant group differences in baseline-corrected spectral power. **(b)** Topographies of group differences for the decrease in beta power (13–30 Hz) are shown across time bins over the time of interest, i.e., disappearance window (−0.3 to 0.7 s). Only bins with significant differences (from cluster-based permutation statistics) are plotted, with black dots indicating significant channels. **(c)** At source level, cluster-based permutation statistics revealed clusters of voxels in bilateral sensorimotor areas showing significant differences between age groups (older-minus-younger) in the beta frequency band which are colored.

To obtain a general overview of the spectral power modulations in the task and to evaluate data quality, we tested the grand average spectral power of all channels against the pre-stimulus baseline window. The analysis was performed using cluster-based permutation statistics in three time-windows that were aligned to (a) the movement onset of the visual stimulus (“movement”), (b) the disappearance of the visual stimulus (“disappearance”), and (c) the reappearance of the visual stimulus (“reappearance”), separately for each group and condition. This revealed significant decreases across a range of different frequencies (all cluster-*p*’s = 0.001; Figure 3a) in the original (Figure 3) and simplified condition (Supplementary Figure 3). After establishing that data quality was satisfactory, we examined age-related differences by comparing baseline-corrected power differences between older and younger adults. Given our primary interest in age-related differences during temporal prediction, we specifically focused on power changes during the disappearance window in which predictions took place. Cluster-based permutation statistics revealed that older adults exhibit larger beta power decreases compared to younger adults, a pattern observed in both the original (cluster-*p = 0*.001, Figures 3a,b) and simplified condition (cluster-*p* = 0.001, Supplementary Figure 3). Importantly, these effects do not seem to be driven by age-related differences at baseline (cluster-*p* > 0.05). No significant differences between groups were found in other frequency bands (all cluster-*p*’s > 0.05). Source level analysis further confirmed a significant decrease of beta power in bilateral sensorimotor areas (including precentral, superior frontal, medial superior frontal, paracentral, postcentral and superior parietal lobes) in older compared to younger adults (cluster-*p* = 0.001, Figure 3c) during temporal prediction. To quantify differences across conditions (original, simplified) in each group, we compared baseline-corrected power differences between the original and simplified condition once for older and once for younger adults. For both groups, cluster-based permutation statistics revealed no significant differences between conditions (all cluster-*p*’s > 0.05, Supplementary Figure 5).

### 3.4 Stronger delta ITPC in older adults compared to younger adults in the original condition

**Figure 4:**
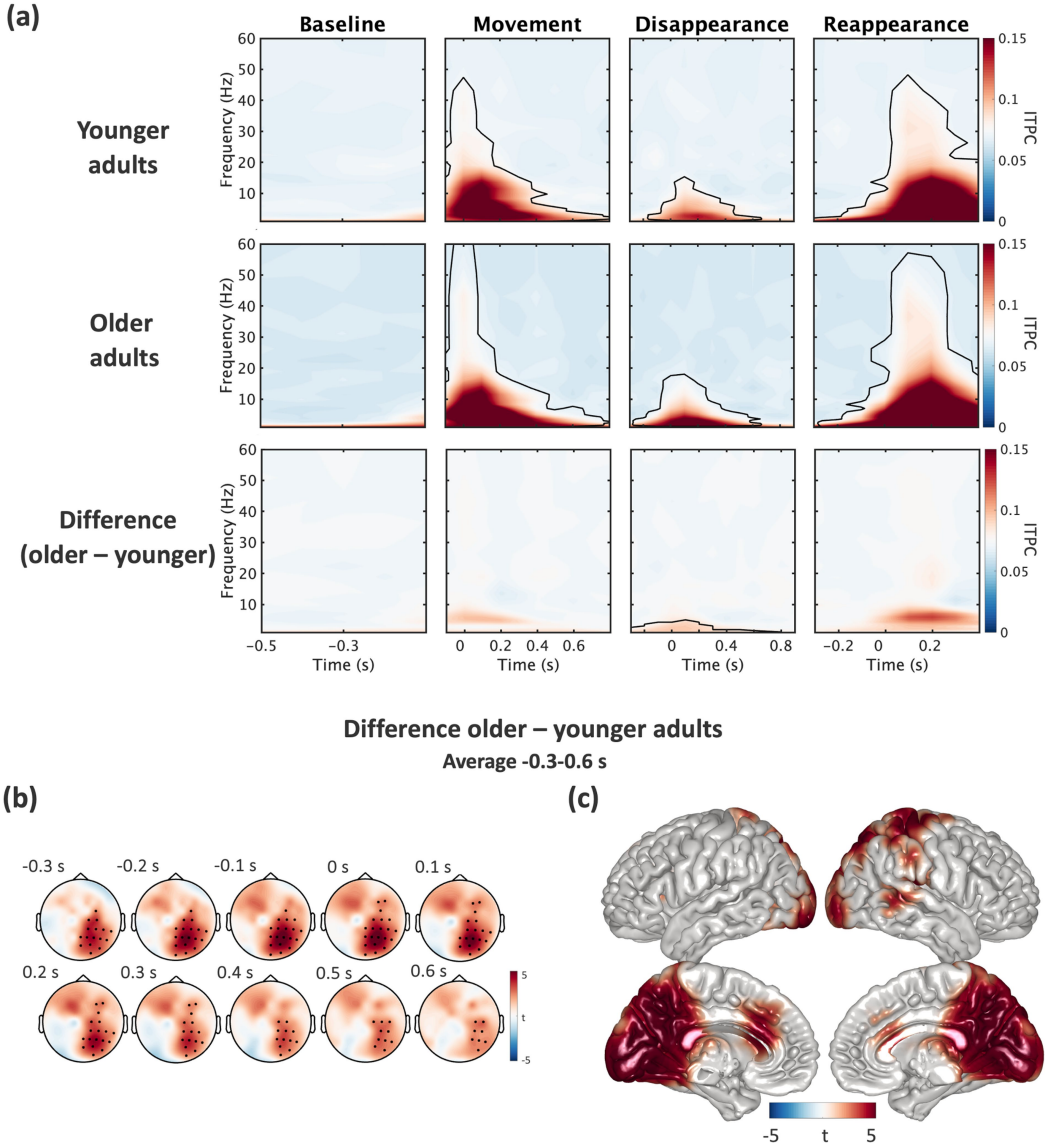
Delta (1–4 Hz) ITPC was stronger in older compared to younger adults. **(a)** ITPC estimates in the original condition are shown separately for younger adults, older adults, and the difference (older minus younger) during the baseline, movement, disappearance, and reappearance window. Regions enclosed by black lines refer to significant ITPC modulations as compared to baseline (cluster-based permutation statistics). **(b)** Topographies for the increase in delta ITPC (1–4 Hz) are showing the difference between both groups (older minus younger) in time bins around the time of interest, i.e., disappearance window (−0.3 to 0.6 s) with black dots indicating significant channels. **(c)** On source-level, cluster-based permutation statistics revealed clusters of voxels showing significant differences between groups (older minus younger) across the occipital, parietal, and right temporal cortex which are colored.

**Figure 5:**
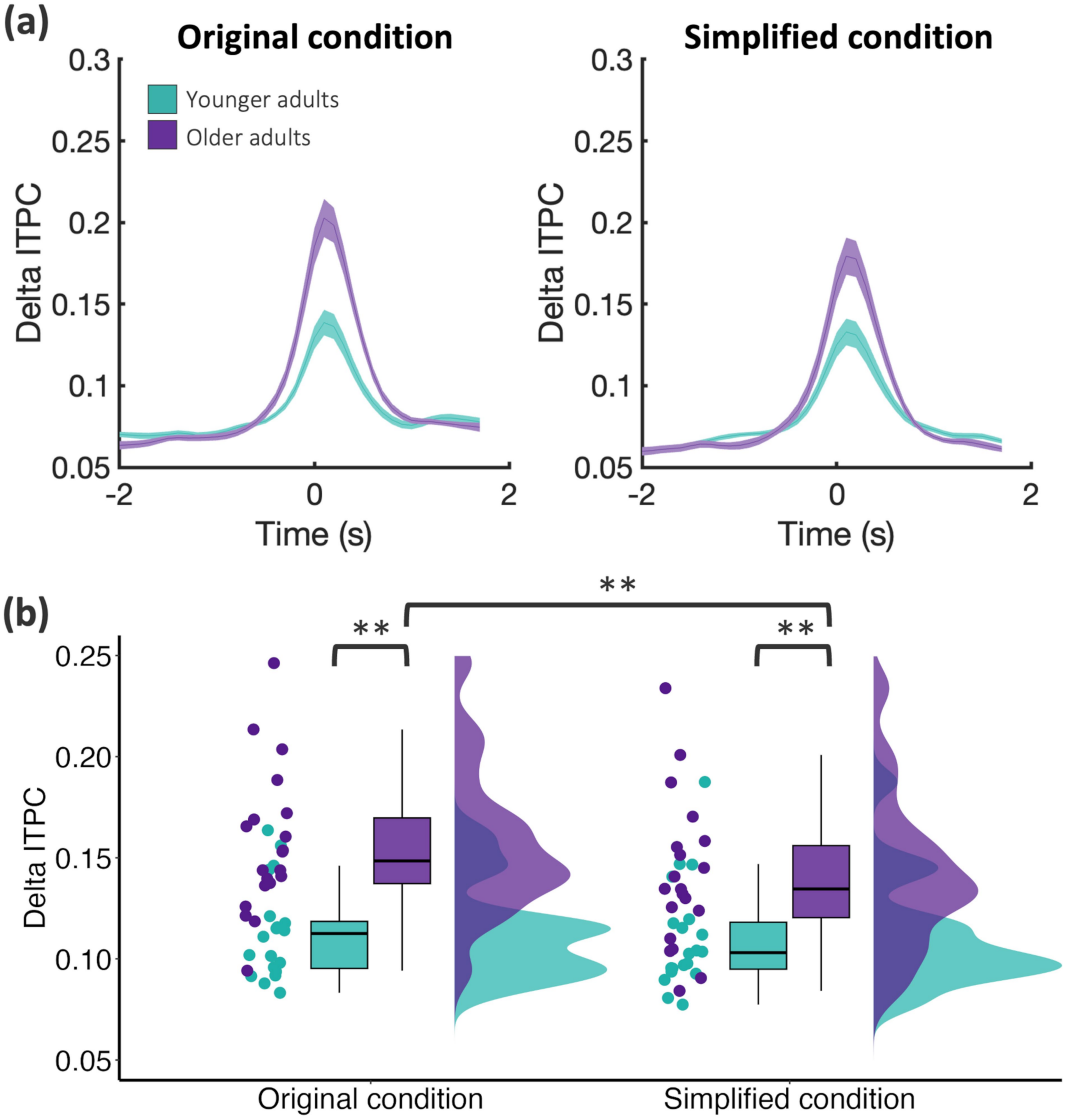
Time course of source-level delta ITPC revealed differences across groups and conditions. **(a)** Time course of delta ITPC (1–4 Hz) was averaged across significant voxels in younger (green) and older adults (purple) in original (left) and simplified (right) condition. **(b)** Delta ITPC was averaged over the disappearance window (−0.3 to 0.6 s) and significant voxels across original (left) and simplified (right) condition in each group. Data are shown on single subject level, as boxplots, and as distributions (each *N* = 20). *p < 0.01.

To obtain a general overview of ITPC estimates associated with performing the temporal prediction task, we tested ITPC differences to baseline in the three time-windows for an average in each condition across all channels using cluster-based permutation statistics. In younger and older adults, ITPC was significantly increased across a range of different frequencies in time windows aligned to movement onset, disappearance and reappearance of the stimulus, respectively (all cluster-*p’*s < 0.001; Figure 4a). Age-related differences in ITPC were then examined by computing the difference between older and younger adults’ ITPC estimates (see Figure 4). Cluster-based permutation statistics revealed a significant increase in ITPC for older adults in the delta frequency band during the disappearance window in the original (cluster-*p* < 0.01; Figures 4b,c, 5) and simplified condition (*p* = 0.01, Figure 5 and Supplementary Figure 4) in a cluster of voxels spanning across the occipital, parietal, and right temporal lobe. To quantify differences across conditions, we compared ITPC estimates between the original and simplified condition separately for older and younger adults. For older adults, cluster-based permutation statistics revealed a significant increase only in delta ITPC for the original compared to the simplified condition in the disappearance window (cluster-*p* < 0.01; Figure 5) suggesting that delta ITPC was enhanced in the original compared to the simplified condition. In younger adults, cluster-based permutation statistics did not reveal any significant differences across conditions.

### 3.5 Temporal prediction performance did not correlate with neural data

**Figure 6:**
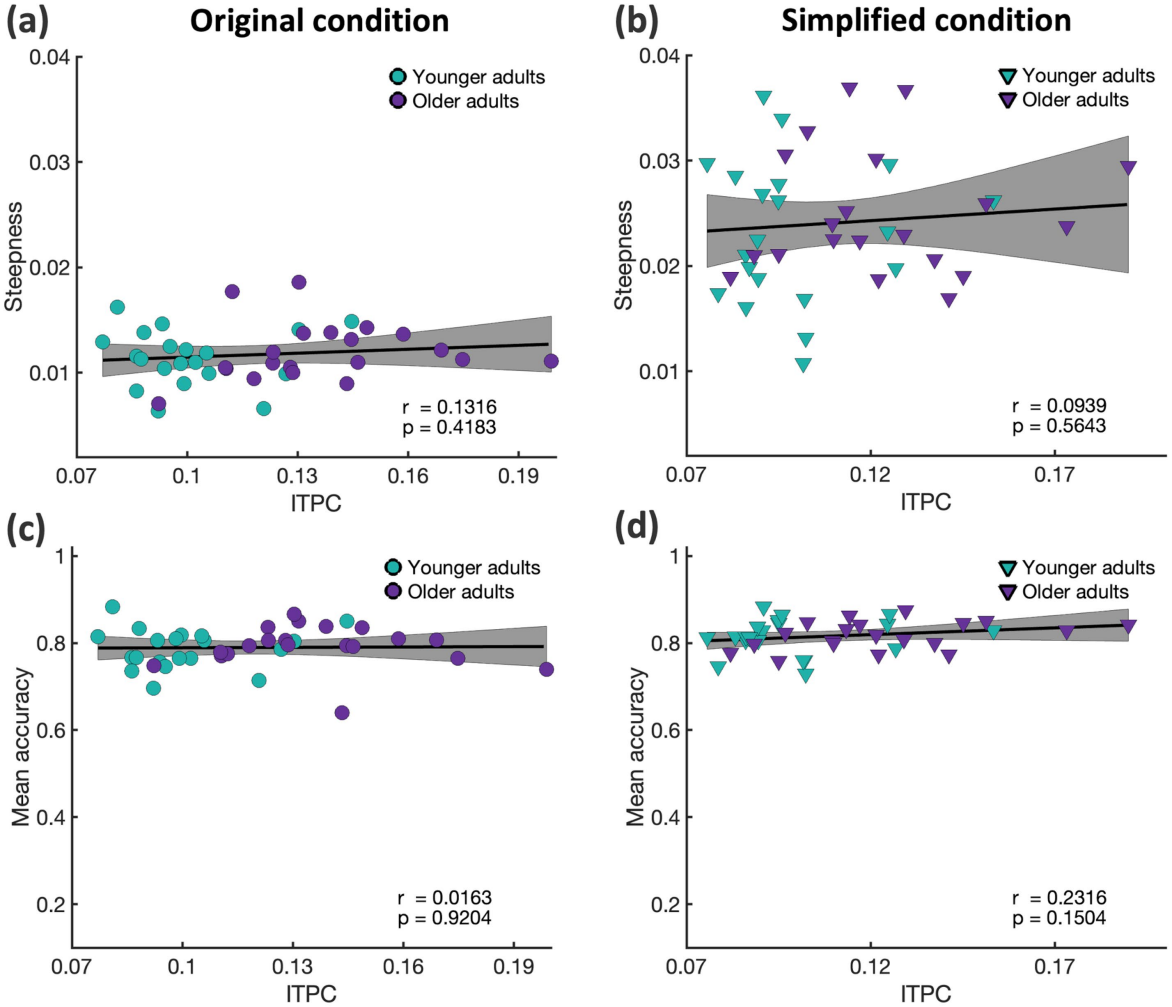
Relationship between individual slope of the psychometric function and source-level delta ITPC in younger (green) and older (purple) adults. ITPC was averaged across the delta frequency band (1–4 Hz) and time window of −0.3 to 0.6 s around the disappearance of the visual stimulus and significant voxels (as indicated from cluster-based permutation statistics see above) for each group and condition separately. Grey area represents the 95% confidence intervals. **(a)** Correlation between slope of the psychometric curve in the original condition and delta ITPC. **(b)** Correlation between slope of the psychometric curve in the simplified condition and delta ITPC. **(c)** Correlation between mean accuracy in the original condition and delta ITPC. **(d)** Correlation between mean accuracy in the simplified condition and delta ITPC.

Building on the idea that phase alignment of neural oscillations supports temporal prediction, we hypothesized that individuals with more precise timing judgements would exhibit stronger ITPC during temporal prediction. Since a steeper psychometric function reflects more consistent responses across trials, we expected participants with steeper functions to show greater ITPC, indicating more stable neural phase across trials. To test this relationship, we computed Pearson correlations between source level delta ITPC and the slope of the psychometric function. However, we did not find any significant correlation (*p* > 0.05 for all clusters, see Figures 6a,b). A similar correlation analysis between delta ITPC and mean accuracy also yielded no significant effects (*p* > 0.05 for all clusters, see Figures 6c,d). To increase statistical power, we repeated these analyses by pooling data across all groups and conditions (*N* = 80), yet no significant correlations emerged (*p* > 0.05 for all clusters). Additionally, we computed the same cluster analysis for correlations between the slope of the curve and source level beta power but found no cluster of voxels showing significant correlations (*p* > 0.05 for all clusters, Supplementary Figure 6).

## 4 Discussion

In this study, we tested whether oscillatory markers of temporal prediction change with ageing by comparing two groups performing a temporal prediction task. Our results indicated comparable temporal prediction performance between younger and older adults, while response times in the task were slower in older compared to younger adults. Notably, older adults exhibited a stronger decrease in beta activity than younger adults, particularly in sensorimotor regions. Further, we observed stronger delta ITPC in occipital and parietal areas in older compared to younger adults. These findings suggest that while temporal prediction performance was maintained across age groups, neural processing differences emerged.

### 4.1 Comparable performance in temporal prediction for younger and older adults

Participants successfully judged timing events throughout the task as indicated by the metrics of the psychometric curve, and in alignment with previous findings (Capizzi et al., 2022; Daume et al., 2021). The main focus of this study was to investigate whether this temporal prediction differed between younger and older adults; however, contrary to our initial hypothesis, temporal prediction performance was comparable in the task. While some research has suggested age-related impairments in predictive timing (Vallesi et al., 2009; Zanto et al., 2011), our findings support recent studies suggesting that explicit timing abilities, such as predicting the reappearance of a stimulus, remain largely intact with age (Chauvin et al., 2016; Droit-Volet et al., 2019; Heideman et al., 2018). Moreover, a systematic evaluation of spatiotemporal predictions across the adult lifespan (20–80 years) showed that although aging is associated with a decline in visual search performance, the ability to leverage predictions to guide attention is largely preserved across the lifespan (Shalev et al., 2024). In light of these findings, several authors concluded that reduced temporal prediction performance in older participants might not reflect a selective temporal prediction deficit but, instead, a decline of other cognitive abilities (Droit-Volet et al., 2019; Shalev et al., 2024). In our study, we carefully screened participants for cognitive impairment using neuropsychological tests and excluded those that failed these tests. Thus, the sample of older adults included in our study consisted of individuals with relatively preserved cognitive function. Alongside the preserved cognitive abilities, our sample had a relatively high education level, potentially contributing to comparable performance across groups (Tari et al., 2023). In addition, the mean age of our sample was slightly above 60 years and, thus, approximately 10 years below the mean age in studies reporting significant age-related declines (Vallesi et al., 2009; Zanto et al., 2011), suggesting that differences may emerge later in life. Therefore, the sample characteristics of participants included in this study may reflect a higher-functioning subgroup of older adults, which could limit the generalizability of the findings. Further work should disentangle temporal prediction from other cognitive abilities potentially leading to age-related differences. Even though we did not observe group differences in temporal prediction performance, performance was significantly better in the simplified condition compared to the original condition as indicated by a steeper slope and higher mean accuracy. This suggests that increasing the reappearance delay facilitated temporal discrimination, making the task easier to perform. Given that temporal prediction deficits are observed in clinical populations such as Parkinson’s disease patients (Tomassini et al., 2019), future studies could explore whether task simplification helps assess predictive timing abilities in these populations. Response times were significantly slower in older adults (see Supplementary Figure 1), a finding consistent with well-established age-related motor slowing (Salthouse, 1996). This slowing is often attributed to greater response caution, reflecting a more deliberate decision-making process rather than a specific deficit in temporal prediction (Lindenberger and Mayr, 2014). Importantly, while response execution was slower, temporal prediction performance remained comparable, suggesting that predictive timing itself was preserved. Together, our results show that both younger and older adults demonstrated the ability to predict time proficiently and clearly indicate that the ability to anticipate timing of events is preserved in normal ageing.

### 4.2 Stronger decrease of beta activity in older compared to younger adults during temporal prediction

Although temporal prediction performance was comparable across age groups, we observed a stronger beta suppression in older compared to younger adults. This pattern was consistent across both the original and simplified conditions of our task, i.e., irrespective of task difficulty. While typically associated with movements (Baker, 2007; Pfurtscheller and da Lopes Silva, 1999), beta activity has also been shown to play a central role in timing-based prediction and thus facilitating the coordination of neural activity to anticipated events (Arnal and Giraud, 2012; de Lange et al., 2013; Donner et al., 2009; Kulashekhar et al., 2016; Saleh et al., 2010). While some studies reported an increase in beta activity (Arnal et al., 2015; Morillon and Baillet, 2017), others, particularly those examining preparatory motor states and sensory prediction, have found that beta activity decreases before expected events, facilitating neural readiness and behavioural responses (Arnal et al., 2015; Daume et al., 2021; Kulashekhar et al., 2016; van Ede et al., 2011). Source localisation of this decrease in beta activity indicated the involvement of sensorimotor areas activity (Arnal et al., 2015; Morillon and Baillet, 2017) and in task-relevant sensory regions such as visual or tactile sensory regions (Daume et al., 2021; van Ede et al., 2011). Thus, the reduction of beta activity during temporal prediction (irrespective of our group effects) we observed in our study, is well in line with the literature.

In ageing, reports on the changes in beta activity during temporal prediction is sparse. Mostly, studies focused on changes in alpha activity and linking age-related declines in temporal prediction to reduced alpha activity (Herrmann et al., 2023; Zanto et al., 2011). While our data clearly comprised alpha band activity, results did not reveal significant group differences in alpha activity when comparing younger and older adults. Instead, we observed a stronger decrease in beta activity in older adults, suggesting that age-related change in predictive processing may be more strongly reflected in motor and timing-related functions rather than attentional suppression. Given that beta oscillations facilitate top-down coordination of sensorimotor processing (Engel and Fries, 2010; Spitzer and Haegens, 2017), older adults may rely more heavily on motor-related beta oscillations to maintain predictive timing than younger adults. Beta suppression has been linked to predictive processing, with stronger suppression potentially indicating a greater reliance on internal models to guide perception in the face of declining sensory acuity (Engel and Fries, 2010). This could imply that older adults recruit additional neural resources to maintain an accurate representation of temporal intervals, compensating for age-related declines in sensory processing.

### 4.3 Stronger increase in delta ITPC in older compared to younger adults

While participants predicted the reappearance of the stimulus, we observed a delta ITPC increase, i.e., phase alignment of delta activity, which was stronger in older compared to younger adults. For older adults, delta ITPC was stronger in the original compared to the simplified condition, while this effect was not present in younger adults. As anticipated, this effect occurred contralaterally to the side of disappearance of the visual stimulus. Neural oscillations have been reported to adjust in phase to optimize sensory selection (Arnal et al., 2011; Schroeder and Lakatos, 2009). Specifically, studies in animals and humans showed that anticipating sensory events resets the phase of delta or delta-theta oscillations before the stimulus occurs, resulting in accelerated response times (Herbst et al., 2022; Lakatos et al., 2008; Stefanics et al., 2010) and improved accuracy (Arnal et al., 2015; Burke et al., 2025; Daume et al., 2021). Delta phase alignment has been proposed to enhance sensory selection by synchronising neural activity to expected events, either through entrainment to rhythmic stimulation (Large and Jones, 1999) or endogenous phase resetting in response to anticipated stimuli (Arnal et al., 2011; Burke et al., 2025; Schroeder and Lakatos, 2009). While entrainment suggests that external cues entrain endogenous rhythms, recent evidence indicates that delta phase resetting can also occur in the absence of rhythmic input (see Daume et al., 2021 for full discussion). Our findings align with this perspective, as delta ITPC was observed even though our task did not include rhythmic stimulation. The stronger delta ITPC observed in older adults suggests that ageing may alter the neural dynamics supporting temporal prediction. Since phase consistency is thought to reflect improved temporal coordination across neural networks (Wilsch et al., 2015), one possibility is that increased phase consistency serves a compensatory function reflecting that older adults rely more heavily on phase resetting to maintain temporal prediction accuracy. The observed increase in delta ITPC in older adults, particularly in the more difficult condition, suggests that ageing may enhance reliance on phase synchronisation to maintain temporal prediction accuracy under increased cognitive demand. However, our results did not reveal a direct correlation between delta ITPC and behavioural accuracy. Therefore, it remains unclear whether stronger phase alignment actually supports performance or reflects a general age-related shift in neural synchronisation. Future studies should examine whether delta ITPC enhancement in aging corresponds to functional improvements or emerges as a by-product of broader age-related neural changes.

### 4.4 Conclusion

We conducted a study to explore whether and how anticipatory oscillatory markers of temporal prediction change with ageing by comparing temporal prediction performance in two age groups. While temporal prediction performance remained comparable between younger and older adults, our findings revealed differences in the neurophysiological patterns with older adults showing a stronger decrease of beta activity and increased delta ITPC during predictive timing. Our findings provide insights into how ageing affects neural dynamics underlying temporal prediction. Although we did not observe a significant relationship between neural measures and behavioural performance, this does not preclude a compensatory interpretation, as such mechanisms may operate at a group level without yielding linear brain-behaviour correlations across individuals. The enhanced beta suppression and delta ITPC in older adults may therefore reflect adaptive neural recruitment supporting preserved performance, although alternative accounts such as neural inefficiency or broader age-related changes in oscillatory dynamics cannot be fully excluded. Future work will be required to more directly establish the functional relevance of these neural differences for behaviour.

## Data Availability

The raw data supporting the conclusions of this article will be made available by the authors, without undue reservation.
