# Supplementary material for: Predicting time across age: comparing performance and neural dynamics of younger and older adults in a temporal prediction task
Source: Front Aging Neurosci. 2026 May 20;18:1790156. doi: 10.3389/fnagi.2026.1790156 (PMC13229979; doi:10.3389/fnagi.2026.1790156)
Supplement: Supplementary file 1 [file Data_Sheet_1.DOCX]

Supplementary Material


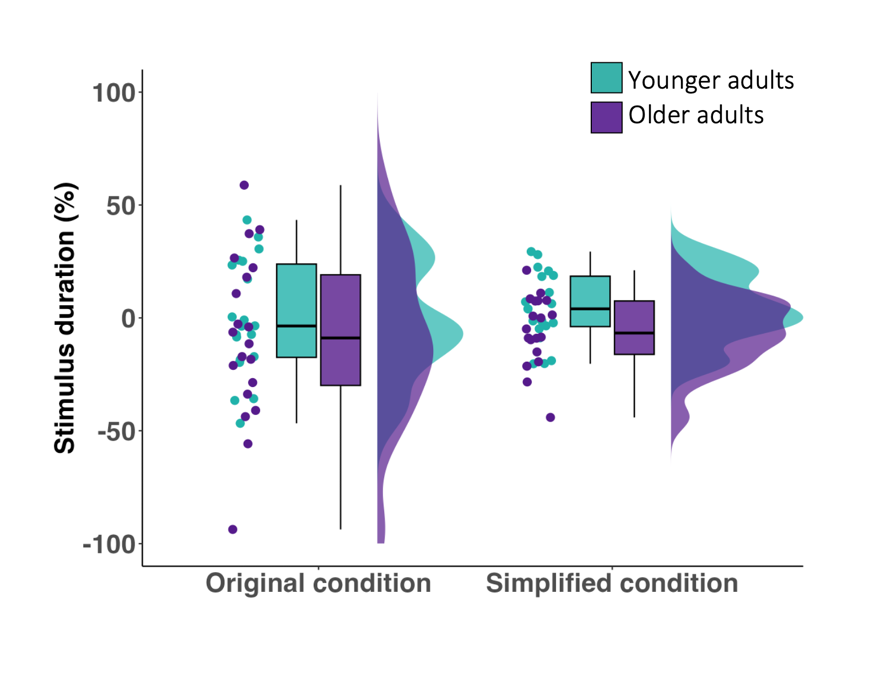


**Supplementary Figure 1.** **Comparable “right on time” for younger (green) and older (purple) adults (each group N=20)** “Right on time” estimates were averaged across original (left) and simplified (right) condition in each group. Data are shown on single subject level, as boxplot, and as distribution (each N=20).


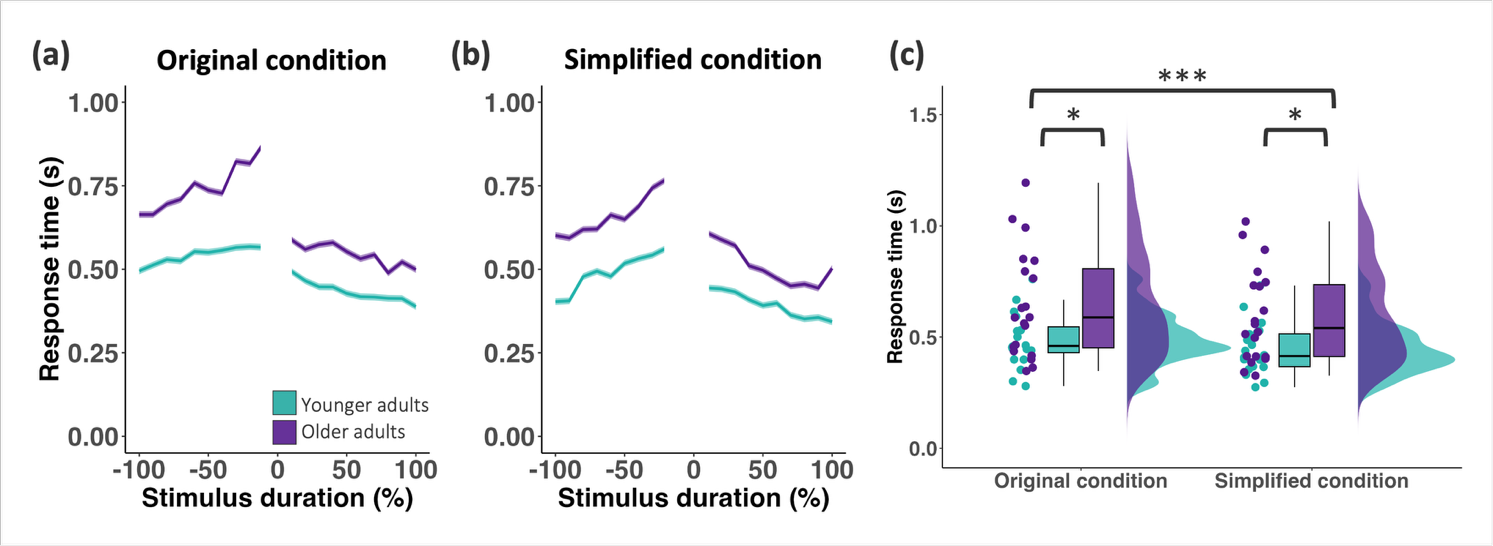


**Supplementary Figure 2. Response times were slower in older adults (purple) compared to younger adults (green) (each group N=20)** Response times are shown across stimulus levels in the original condition **(a)** and simplified condition **(b)**. **(c)** Response times were averaged across the original (left) and the simplified (right) condition in each group. Data are shown on single subject level, as boxplots, and as distributions (N=20). * p < .05, *** p < .001.

**
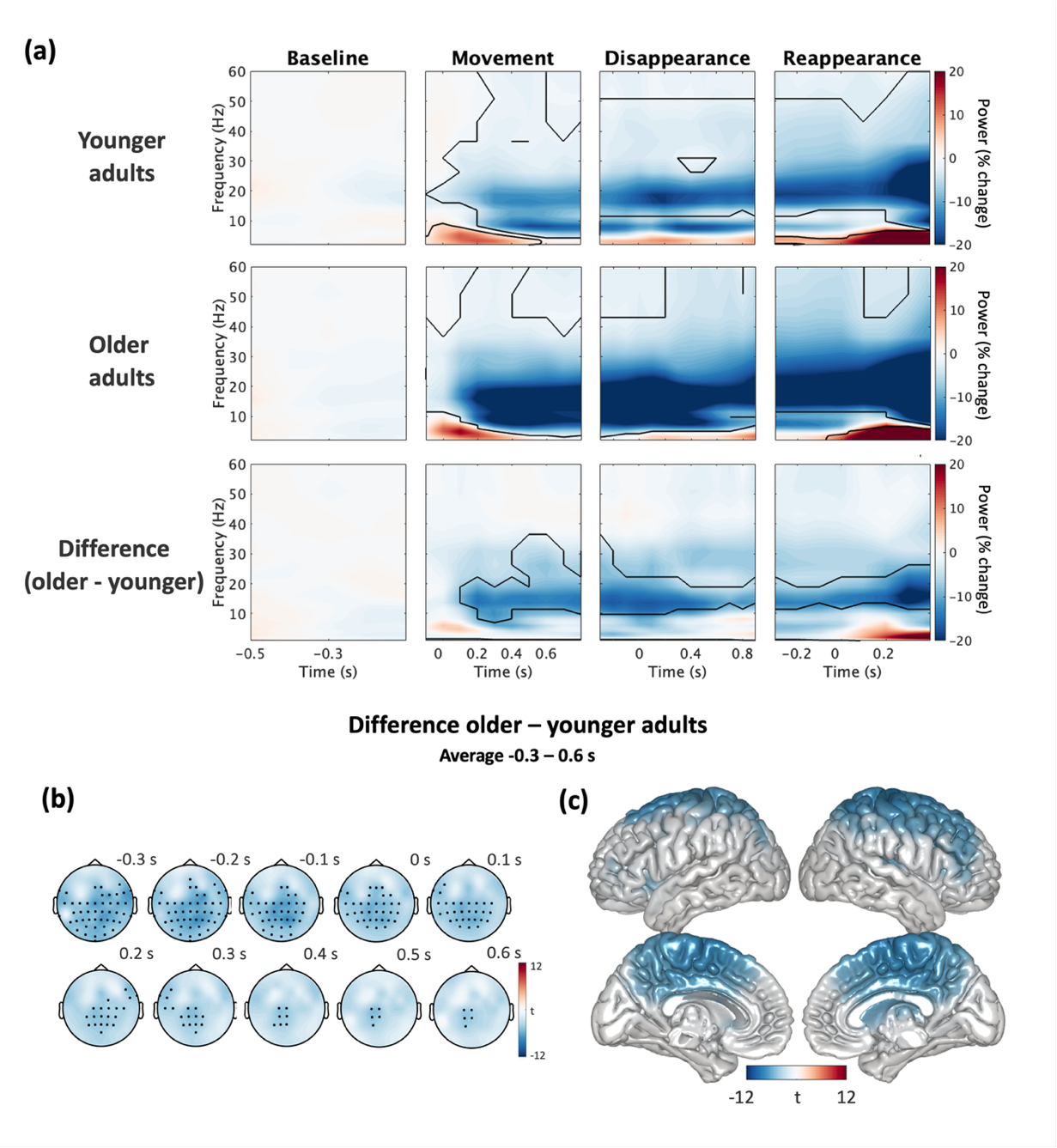
**

**Supplementary Figure 3. Grand-average power modulation in the simplified condition of the temporal prediction task**

**(a)** Spectral power dynamics in the simplified condition are shown separately for younger adults, older adults and the difference between both groups (older-minus-younger adults) during the baseline, movement, disappearance and reappearance window of the temporal prediction task. For each window, epochs were aligned to the respective events (time 0 s). Spectral power was baseline-corrected within each group. Cluster-based permutation statistics revealed significant power modulations as compared to baseline (highlighted by continuous lines) and significant group differences in baseline-corrected spectral power.

**(b)** Topographies of group differences for the decrease in beta power (13-30 Hz) are shown across time bins over the time of interest, i.e., disappearance window (-0.3 to 0.6 s). Only bins with significant differences (from cluster-based permutation statistics) are plotted, with black dots indicating significant channels.

**(c)** At source level, cluster-based permutation statistics revealed clusters of voxels in bilateral sensorimotor areas showing significant differences between age groups (older-minus-younger) in the beta frequency band which are coloured.

**
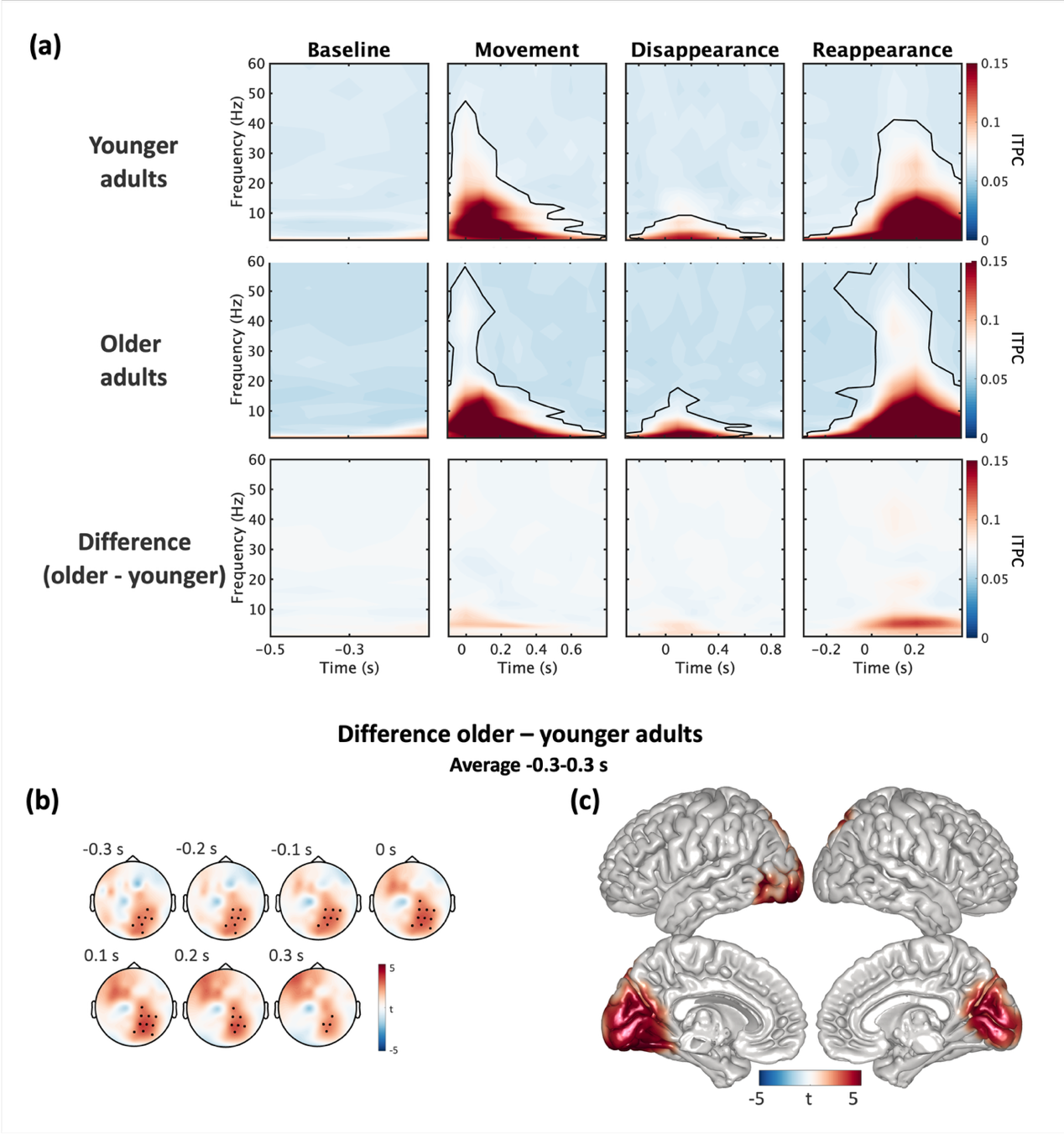
**

**Supplementary Figure 4. Delta (1-4 Hz) ITPC was stronger in older compared to younger adults in the simplified condition**

**(a)** ITPC estimates in the simplified condition are shown separately for younger adults, older adults and the difference between groups (older-minus-younger) during the baseline, movement, disappearance and reappearance windows. Regions enclosed by black lines refer to significant ITPC modulations as compared to baseline (cluster-based permutation statistics).

**(b)** Topographies for the increase in delta ITPC (1-4 Hz) are showing the difference between both groups (older-minus-younger) in time bins around the time of interest, i.e., disappearance window (-0.3 to 0.3 s) with black dots indicating significant channels.

**(c)** On source-level, cluster-based permutation statistics revealed clusters of voxels showing significant differences between groups (older-minus-younger) across the occipital, parietal, and right temporal lobe which are coloured.


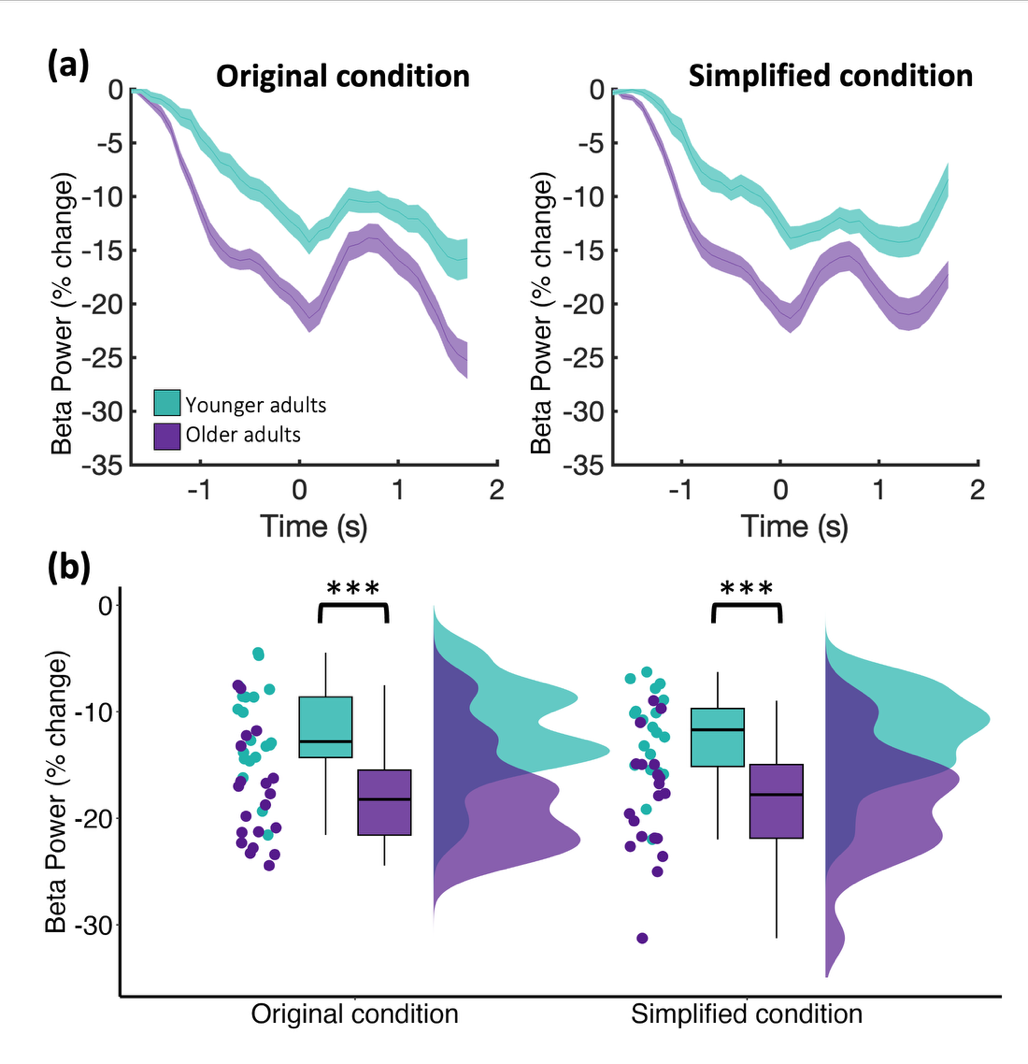


**Supplementary Figure 5. Time course of source-level beta power revealed differences across groups**

**(a)** Time course of beta power (13-30 Hz) was averaged across significant voxels in younger (green) and older adults (purple) in original (left) and simplified (right) condition.

**(b)** Beta power was averaged over the Disappearance window (-0.3 to 0.7 s) and significant voxels across original (left) and simplified (right) condition in each group. Data are shown on single subject level, boxplot, and distribution (each N=20). *** p < .001.


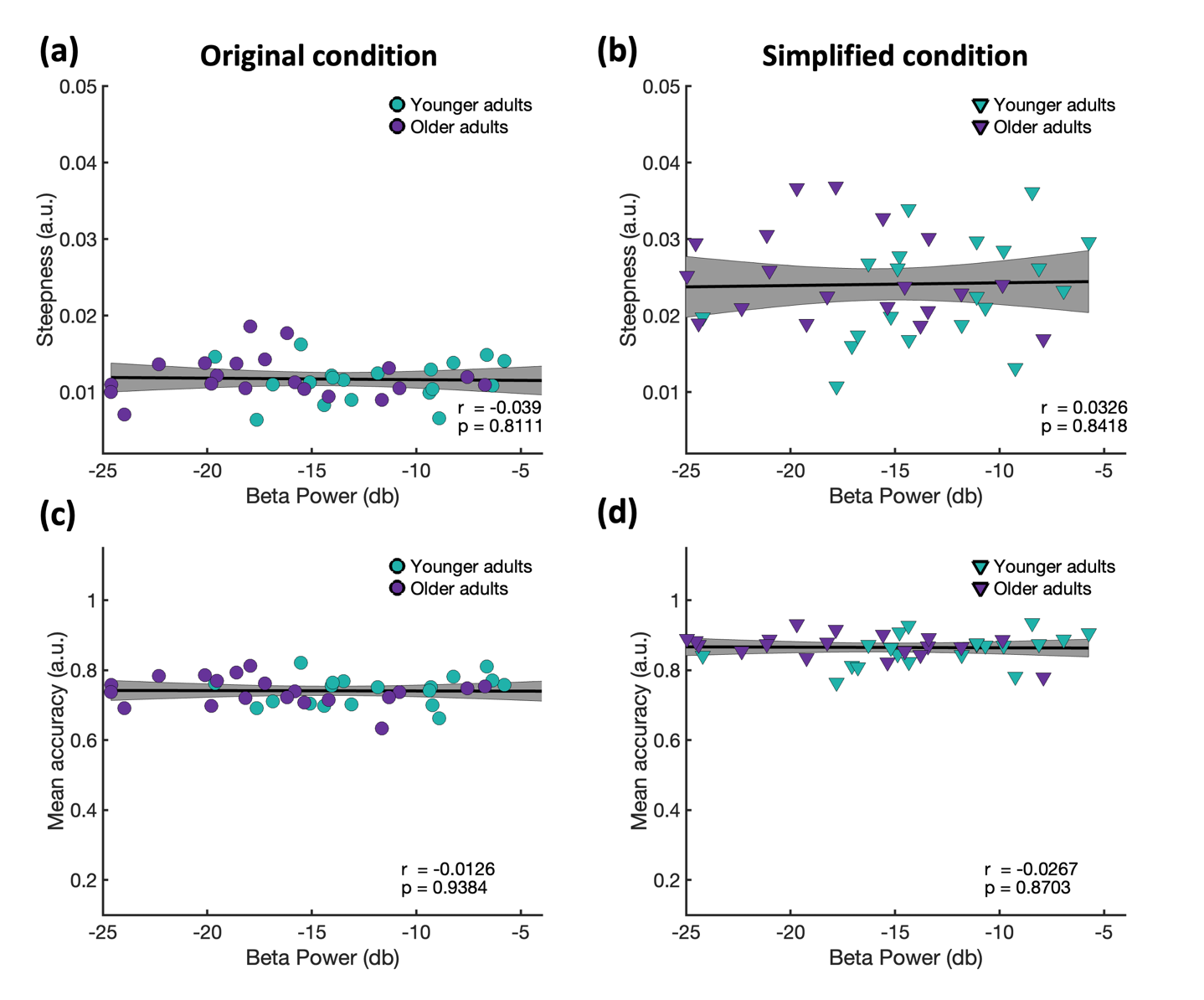


**Supplementary Figure 6. Relationship between individual slope of the psychometric function and source-level beta power in younger (green) and older (purple) adults**

Beta power was averaged across the beta frequency band (13-30 Hz) and time window of -0.3 to 0.7 s around the disappearance of the visual stimulus and significant voxels (as indicated from cluster-based permutation statistics see above) for each group and condition separately. Grey area represents the 95% confidence intervals.

**(a**) Correlation between slope of the psychometric curve in the original condition and beta power.

**(b)** Correlation between slope of the psychometric curve in the simplified condition and beta power.

**(c)** Correlation between mean accuracy in the original condition and beta power.

**(d)** Correlation between mean accuracy in the simplified condition and beta power.
